# Integrative Machine Learning of Glioma and Coronary Artery Disease Reveals Key Tumour Immunological Links

**DOI:** 10.1111/jcmm.70377

**Published:** 2025-01-27

**Authors:** Youfu He, Ganhua You, Yu Zhou, Liqiong Ai, Wei Liu, Xuantong Meng, Qiang Wu

**Affiliations:** ^1^ Medical College Guizhou University Guiyang Guizhou Province China; ^2^ Department of Cardiology Guizhou Provincial People's Hospital Guiyang Guizhou Province China; ^3^ Department of Research The Second People's Hospital of Guizhou Province Guiyang Guizhou Province China; ^4^ Office of Student Affairs Guiyang Healthcare Vocational University Guiyang Guizhou Province China; ^5^ Department of Pathology Army 79th Group Hospital Liaoyang Liaoning Province China

**Keywords:** genetic biomarkers, glioma, immunotherapy, machine learning, precision oncology, single‐cell RNA sequencing, tumour microenvironment

## Abstract

It is critical to appreciate the role of the tumour‐associated microenvironment (TME) in developing strategies for the effective therapy of cancer, as it is an important factor that determines the evolution and treatment response of tumours. This work combines machine learning and single‐cell RNA sequencing (scRNA‐seq) to explore the glioma tumour microenvironment's TME. With the help of genome‐wide association studies (GWAS) and Mendelian randomization (MR), we found genetic variants associated with TME elements that affect cancer and cardiovascular disease outcomes. Using machine learning techniques high dimensional data was analysed to obtain new molecular sub‐types and biomarkers that are important for prognosis and treatment response. F3 was identified as a top regulator and revealed potential angiogenic and immunogenic characteristics within the TME that could be harnessed in immunotherapy. These results demonstrate the potential of machine‐learning approaches in identifying and dissecting TME heterogeneity and informing treatment in precision oncology. This work proposes improving the immunotherapeutic response through targeted modulation of relevant cellular and molecular interactions.

## 1 Introduction

Compared with other diseases, such as glioma and Coronary heart disease (CHD), two most prevalent health challenges worldwide due to their complicated etiologies [1, 2]. Glioblastoma (GBM) or gliomas are the most aggressive and incurable form of brain tumours originating from glial cells, well known for their high rate of progression after post‐surgery tumour resection [3]. Although substantial surgery combined with radiotherapy and chemotherapy has been accomplished, the average survival time of patients diagnosed as glioma highest malignancy (glioblastoma) is still very short in 5 months [4]. In CHD—another ischaemic heart disease but this time due to coronary atherosclerosis—a substantial morbidity and mortality [5] exists. The pathogenesis of EC is intricate and diverse, leading to numerous factors abounding in immunity [5]. Its pathogenesis is complex and varied, and is related to various factors, including immunity, tumour, inflammation and metabolism [6]. Both conditions exhibit substantial genetic heterogeneity, which complicates our understanding and treatment of these diseases.

With the recent progress in genomics and bioinformatics, our ability to study the genetic component of complex diseases has greatly improved. GWAS have played a major role in discovering DNNs connecting genetic variants and diseases, such as glioma [7]. For example, although GWAS can identify potentially pathogenic variants the extent to which they define gene function is also limited. Single‐cell RNA sequencing (scRNA‐seq) is an important frontier to address this gap: scRNA‐seq can infer cellular heterogeneity and reveal the gene expression patterns at unwonted resolutions [8]. Single‐cell gene expression analyses enable researchers to dissect the heterogeneity of cellular phenotypes that underlie pathogenesis. Concurrently, machine‐learning approaches have reshaped the landscape of data analysis in biomedicine. Using large datasets machine learning algorithms can detect complex patterns and associations that are usually not easily revealed by traditional statistical methods [9]. When combined with genetic and transcriptomic data, machine learning can enhance our ability to predict disease outcomes and identify potential therapeutic targets.

Here, we combine genetic inference from GWAS with single‐cell expression analysis to identify and validate putative gene regulatory targets in CHD and brain cancer (glioma). In particular, we concentrate on the F3 gene that have exhibited potential links to these diseases. We use Mendelian randomization (MR) methods to infer causality between these genetic targets and disease phenotypes [10]. We also explored the expression of these genes in various cell types with help from scRNA‐seq data and investigated their function by performing in vitro experiments.

We leverage state‐of‐the‐art genomic technologies and computational methods in our integrated study to provide comprehensive views of the molecular mechanisms driving glioma and CHD. These insights from the research could lead to new specific treatments that may provide better clinical relief for patients with these two devastating diseases.

## 2 Methods

### 2.1 Study Design

Conducting randomised controlled trials (RCTs) is difficult in clinical practice. MR, however, provides a useful genetic epidemiological alternative for discovering and quantifying causal effects. MR looks at genetic variation, which is used as an instrumental variable (IV) in MR analysis. And, critically the sequence is defined: genetic variation precedes trait development. It is necessary to select genetic variations that are strongly correlated with exposure factors. These variations are typically identified as single nucleotide polymorphisms (SNPs) through genome‐wide association studies (GWAS). One of the criteria for selection is that the association between these SNPs and exposure factors must reach a genome‐wide significance level, with a *p* < 5 × 10^−8^. The selected genetic variation should be independent of any confounding factors that may affect the exposure outcome association. This means that genetic variation should not be directly related to outcomes, except through exposure factors in practical operation, it is also necessary to consider the linkage imbalance between genetic variations. If the LD between two SNPs is too high (usually measured as LD > 0.2), one of them needs to be removed to avoid multicollinearity Use a relaxed *p*‐value threshold to filter IVs, such as 5 × 10^−5^, especially in specific situations or to increase the statistical power of the analysis in order to verify the reliability and stability of the results, in addition to the main MR analysis methods (such as IVW), sensitivity analysis such as MR Egger regression is also used.

### 2.2 GWAS Database

Reporting of this study conform to the STROBE‐MR guidelines [11]. The datasets used for this MR analysis were sourced entirely from publicly available genome‐wide association study (GWAS) summary data, including four related to coronary heart disease (CHD) and one on glioma, as detailed in Table 1. We used UK Biobank (gwas.imatorsendphpys glGen9994) for data on exposed populations (mrcieu.ac.uk/datasets). For the glioma data, we utilise all of (predominantly) Finnish population from Finland database; similarly for CHD this is European‐only individuals. For the CHD data, a fixed‐effects meta‐analysis was conducted.

**TABLE 1:** GWAS data and sources.

Exposure/Outcome	Data sources	Population	Sample size	SNPs	Year	GWAS ID	Author	n case	n control
Coronary heart disease	IEU	European	184,305	9,455,779	2015	ieu‐a‐7	Nikpay	60,801	123,504
Coronary artery disease (firth correction)	GWAS catalogue	European	352,063	11,027,870	2021	ebi‐a‐GCST90013864	Mbatchou J	29,338	322,725
Coronary artery disease	GWAS catalogue	European	547,261	7,934,254	2017	ebi‐a‐GCST005195	van der Harst P	122,733	424,528
Coronary artery disease	GWAS catalogue	European	296,525	7,904,237	2017	ebi‐a‐GCST005194	van der Harst P	34,541	261,984
Brain glioblastoma	FinnGen biobank	European	218,792	16,380,466	2021	finn‐b‐C3_GBM	NA	91	218,701

### 2.3 Single‐Cell Analysis

These checks verify acceptable RNA counts, standard for the field. Data are pre‐processed, including cell quality filtering to remove low‐quality cells and removal of unwanted batch effects (e.g., ambient RNA). We set the threshold for the proportion of mitochondrial gene expression count to 15%, the threshold for UMI in cells to 500, and the threshold for the number of detected genes to 250. Forms part‐preprocessing and PCA (principal component analysis) to characterise self‐similarity patterns within cell types or states. We then made PCA scatter plots shows a distribution of tumour samples corresponding to top principal components. This is followed by use of t‐SNE (t‐distributed Stochastic Neighbour Embedding) to cluster cells, separate cell populations and endpoint plots for clustering show variety in cell type or some potential subtypes. Cell–cell interaction network graphs are generated to explore the frequency of interactions between myeloid cells, TILs (T cells—red; NK and NKT populations combined), endothelial fibroblasts stromal proliferating dendritic (B IL‐3Rdendritic cills type 1 ceus population) highlighting key cellular pathways involved in signal transduction.

### 2.4 GO/KEGG Analysis

When utilising R packages like limma or DESeq2 to identify differentially expressed genes (DEGs) from RNA‐Seq data, ensure that the results include gene identifiers (such as Entrez IDs or gene symbols) along with their respective statistical measures. The clusterProfiler R package is designed to facilitate this process, focusing on functional enrichment analysis. It covers gene ontology (GO) categories, which include biological process (BP), molecular function (MF) and cellular component (CC). For the enrichment analysis of KEGG pathways, the function enrichKEGG is employed.

### 2.5 Machine Learning Algorithm

In a machine learning project, we first use the createDataPartition function from the caret package to split the dataset into training and testing sets, typically with a 50% training and 50% testing ratio. This ensures the model has enough data for training while reserving a portion for final evaluation. Next, we use the train function to train various machine learning models, setting cross‐validation parameters with trainControl, such as 10‐fold cross‐validation, to optimise model parameters and assess performance. During model training, we perform grid search using the tuneGrid parameter to select appropriate parameters, like the regularisation parameter C and kernel type for SVMs. After training, we use the explain function from the DALEX package to interpret each model and the predict function along with ROC curves to evaluate model accuracy on the test set. Additionally, the variable_importance function in DALEX helps calculate the importance of variables in each model, providing insights into feature contributions. For Lasso regression, we use the glmnet package, allowing us to control model complexity through regularisation. These steps enable us to build machine learning models that are both accurate and interpretable.

### 2.6 Immunomodulatory Molecules

Immunomodulatory molecules are critical for cancer immunotherapy and many immunomodulatory molecule agonists and antagonists are being evaluated in clinical oncology. We investigated the expression of immunoregulatory molecules and epigenetic control of expression.

### 2.7 Analysis of Immune Cells

To ensure the quality and consistency of the data, immune infiltration data for all TCGA samples were collected from the publicly available database TIMER 2.0. The Spearman correlation coefficients obtained from the analysis were fully visualised in heatmaps in order to visualise the relationship between different cell types and MS4A6A expression under different algorithms. We used the Bonferroni correction method to adjust the *p*‐value to control the risk of Type 1 errors (false positives). In addition, we also provide the analysis results of FDR (false discovery rate) correction for readers' reference. These Supporting Information help improve the transparency and reliability of research methods.

### 2.8 Exploration of Targeted Drugs

To investigate potential therapeutic options to counteract gene‐mediated tumour promotion, we conducted cMAP analysis [12, 13]. We developed a gene‐associated signature comprising the 150 most significantly up‐regulated and 150 most significantly down‐regulated genes by comparing tumours with high and low gene expression.

## 3 Results

### 3.1 Identifying the Targets Indicating the Causal Relationship Between CHD and Glioblastoma Multiforme (GBM) Patients

**FIGURE 1:**
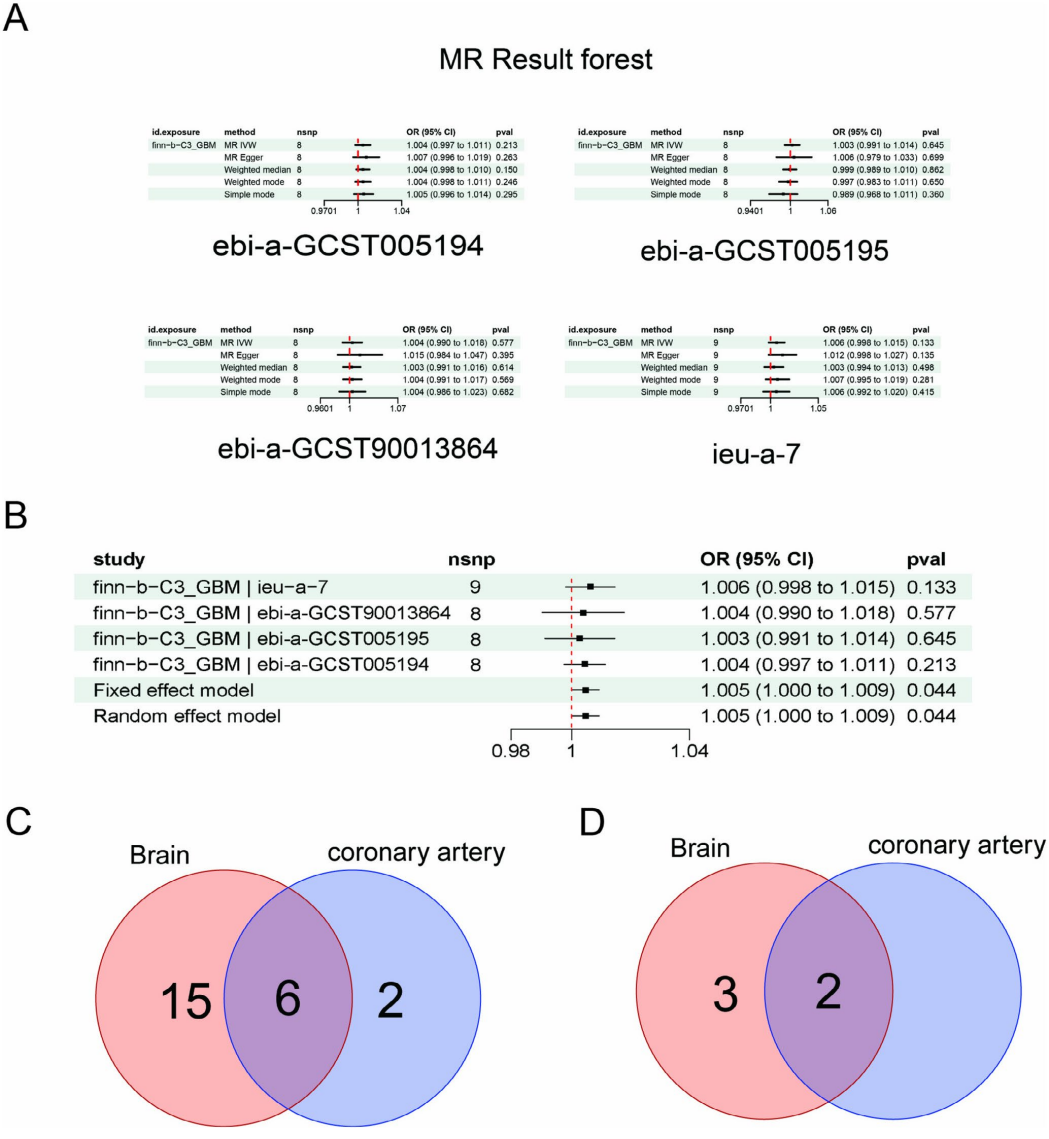
Exploring the causal relationship between coronary heart disease (CHD) and glioblastoma multiforme (GBM) based on Mendelian randomization analysis.

**FIGURE 2:**
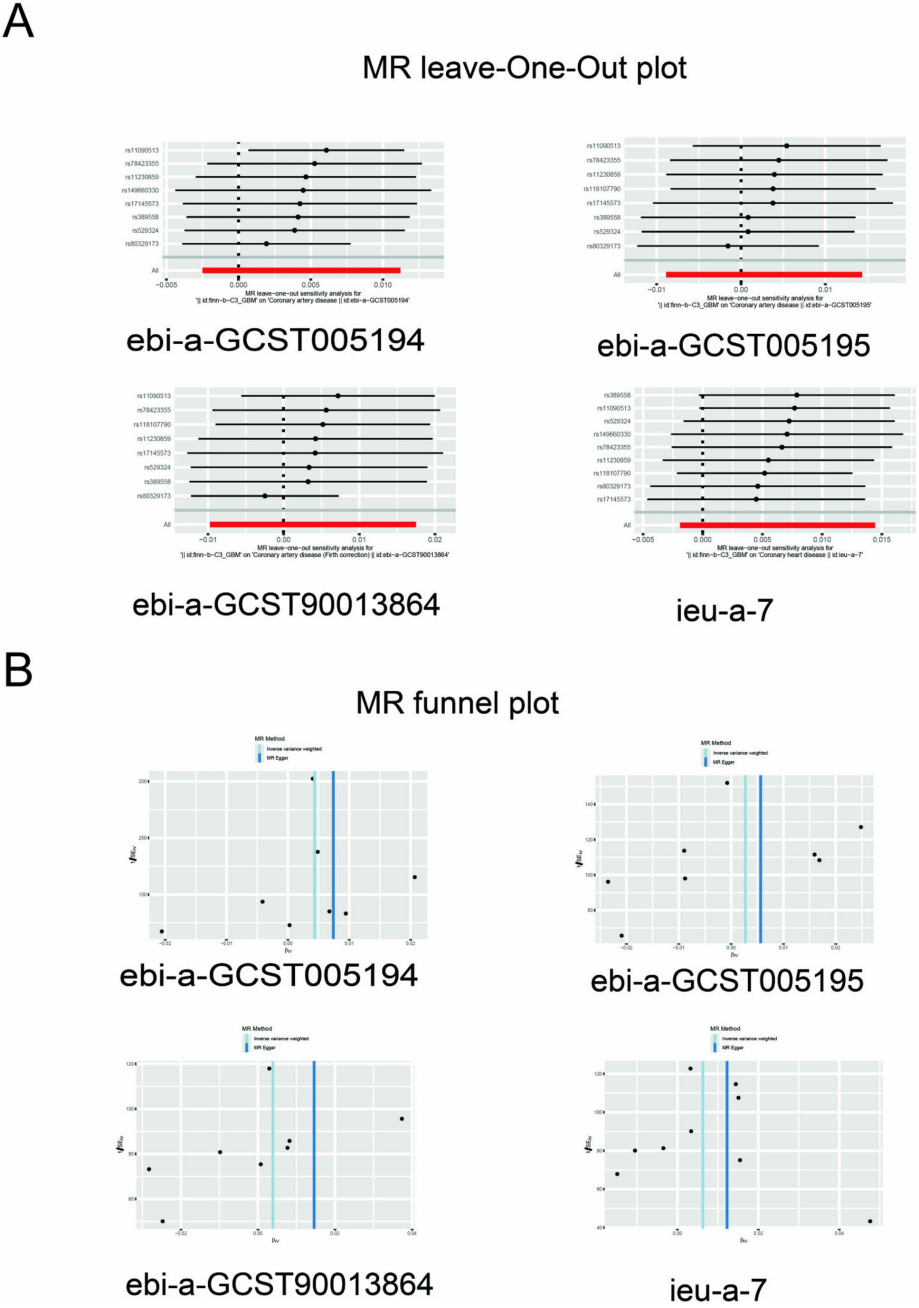
MR leave‐one‐out plot and MR funnel plot for CHD and GBM. (A) MR leave‐one out plot. (B) MR funnel plot.

In this part of the study, we selected patients with GBM multiforme from Finnish data as a phenotype for the disease Glioma. In order to identify potential causal relationships between CHD and GBM, a MR analysis was performed (Figure 1A). Subsequently, the MR results were subjected to meta‐analysis in order to provide further confirmation of the mutual causality. The results of meta‐MR analyses (Figure 1B) showed a significant positive causal relationship between GBM and CHD, with each standard deviation increase in this exposure to GBM also increasing the risk of CHD (OR = 1.005; 95% CI = 1.000–1.009; *P*‐meta = 0.044). We then combined tissue expression Quantitative Trait Loci (eQTL) data from the summary‐data‐based Mendelian randomization (SMR) database for SMR analysis. The brain tissues included in the SMR analysis contained 13 tissue sources, namely Brain_Cerebellar_Hemisphere, Brain_Cerebellum, Brain_Cortex, Brain_Frontal_Cortex_BA9, Brain_Hypothalamus, the Brain_Amygdala, Brain_Anterior_cingulate_cortex_BA24, Brain_Caudate_basal_ganglia, Brain_Hippocampus, Brain_Nucleus_accumbens_basal_ ganglia, Brain_Putamen_basal_ganglia, Brain_Spinal_cord_cervical_c‐1, Brain_Substantia_nigra. eQTL data from one coronary artery tissue was also included for co‐analysis. SMR analysis (Table S1, Figure 1C) showed that 21 genes from 13 regions of brain tissue were associated with GBM, and eight genes from the coronary arteries were associated with GBM. Six of these genes are co‐expressed in both tissues. After screening by HEIDI test (Figure 1D), only two genes were finally co‐expressed in both tissues, TNS2‐AS1 (*P*‐SMR = 0.0009, *P*‐HEIDI = 0.6224) and RP11‐463O9.9 (*P*‐SMR = 0.0007, *P*‐HEIDI = 0.3864). The spatial distribution of the two genes on specific chromosomes is shown in Figure 1E,F. The main leave‐one‐out analysis result plots and funnel plots in the MR analysis are shown in Figure 2A,B. All the remaining heterogeneity, horizontal pleiotropy and Directionality test are shown in Table 2.

**TABLE 2:** Heterogeneity, pleiotropy and directionality tests.

id.exposure	Heterogeneity test				Pleiotropy test		Directionality test	
	MR Egger		IVW		MR Egger		steiger	
	*Q*	*Q_P*	*Q*	*Q*_*P*	egger_intercept	*p*	correct_causal_direction	*p*
ieu‐a‐7	8.85	0.264	10.14	0.26	−0.01	0.35	TRUE	0.87
ebi‐a‐GCST90013864	17.7	0.007	9.4	0.01	−0.01	0.48	TRUE	0.859
ebi‐a‐GCST005195	24.3	0.000	24.5	0	−0	0.82	TRUE	0.85
ebi‐a‐GCST005194	17	0.009	18.1	0.01	−0.01	0.54	TRUE	0.871

Two sample MR analysis (A): Forest plot of MR analysis results validated using five methods. Where exposure is GBM and outcomes are four different CHD data. Meta‐analysis (B): Forest plot of meta‐analysis of the results of the four MR analyses. Venn diagram (C, D): The results of SMR analyses from brain tissue, as well as SMR analyses from coronary tissue, were subjected to Wayne's analysis. Figure C shows the data for total differentially expressed genes, and Figure D shows the data after performing the HEIDI test and screening for *p* > 0.05.

### 3.2 A Comprehensive Analysis of Gene Expression and Its Impact on Survival in GBM Patients

**FIGURE 3:**
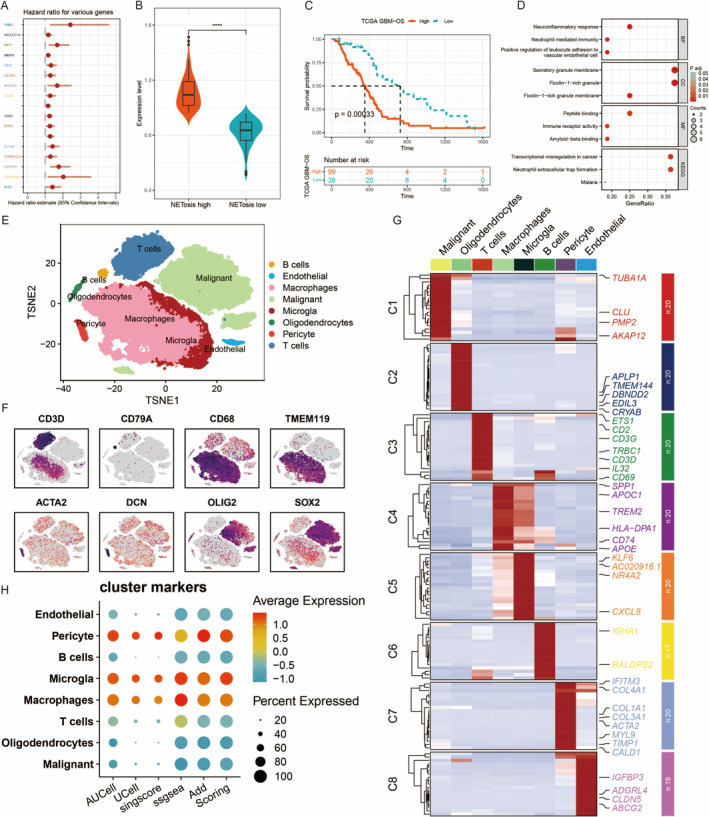
A comprehensive analysis of gene expression and its impact on survival in GBM patients. Hazard ratios (A): This panel likely shows the association between various genes and patient survival, indicating which genes might be linked to prognosis. Violin plot (B): Displays the expression levels of a specific gene or gene set, comparing high and low expression groups, which may correlate with clinical outcomes. Survival Analysis (C): Kaplan–Meier curve demonstrating survival differences between groups with high and low expression of a particular gene or signature. Pathway analysis (D): Enrichment analysis highlighting pathways significantly associated with the gene expression data, indicating potential biological processes involved in glioma. t‐SNE plot (E): Visualisation of cell clusters based on gene expression, identifying distinct cell types such as T cells, macrophages and malignant cells within the tumour microenvironment. Gene expression maps (F): Spatial distribution of specific marker genes across different cell types, providing insights into their roles and locations within the tissue. Heatmap (G): Displays gene expression levels across various cell clusters, highlighting differentially expressed genes that may play roles in glioma biology. Dot plot (H): Illustrates the average expression and percentage of cells expressing cluster marker genes, offering a summary of cell type‐specific expression patterns.

This panel displays the hazard ratios for different genes, indicating their impact on patient survival. The hazard ratio (HR) and 95% confidence intervals (CIs) are shown, where an HR > 1 suggests a higher risk of adverse outcomes associated with the gene's expression (Figure 3A). This violin plot compares the expression levels of a specific gene between two groups: high and low NETosis. The width of the plot represents the density of the data points, and the box plot inside shows the median and interquartile range. The significant difference between the groups is indicated by the asterisks (****) (Figure 3B). This Kaplan–Meier curve depicts the overall survival (OS) of patients stratified by high and low expression of a particular gene. The *p*‐value (*p* = 0.00033) indicates a significant difference in survival between the two groups, with the number of patients at risk shown below the plot (Figure 3C). This dot plot represents the enrichment of various GO terms in the analysed dataset. The size of the dots indicates the number of genes associated with each GO term, whereas the colour gradient represents the significance level (adjusted *p*‐value) (Figure 3D). The t‐SNE plot shows the clustering of single cells based on their gene expression profiles. Different cell types are colour‐coded, including B cells, endothelial cells, macrophages, microglia, oligodendrocytes, pericytes, T cells and malignant cells. This visualisation helps identify distinct cell populations within the dataset (Figure 1E). These plots display the expression levels of specific marker genes (CD3D, CD79A, CD68, TMEM119, ACTA2, DCN, OLIG2 and SOX2) across the t‐SNE clusters. The intensity of the colour represents the expression level, highlighting the distribution of these markers in different cell types (Figure 3F). The heatmap shows the expression levels of selected genes across different cell clusters (C1–C7). Each row represents a gene, and each column represents a cell cluster. The colour gradient indicates the expression level, with red representing high expression and blue representing low expression. The dendrogram on the left clusters genes with similar expression patterns (Figure 3G). This dot plot illustrates the average expression and percentage of cells expressing specific marker genes in different cell types (endothelial, pericyte, B cells, microglia, macrophages, T cells, oligodendrocytes and malignant) (Figure 3H).

### 3.3 A Comprehensive View of Gene Expression and Cell–Cell Interactions in the Context of Glioma

**FIGURE 4:**
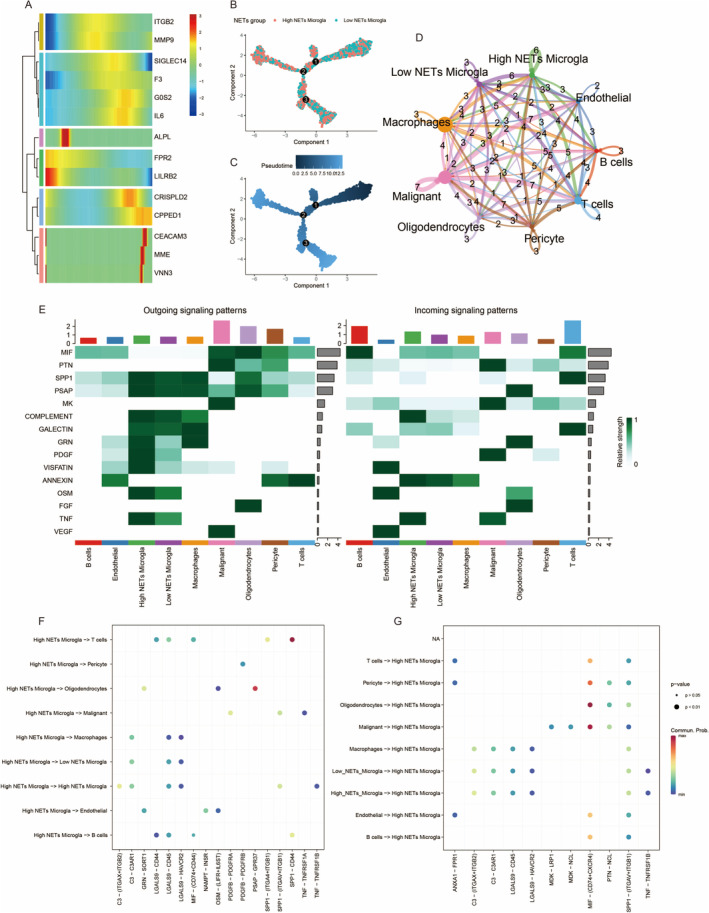
A comprehensive view of gene expression and cell–cell interactions in the context of glioma. (A) Heatmap of gene expression across cell types, highlighting genes like F3. (B) UMAP plot showing high and low NETs microglia subpopulations. The colour coding represents different cell populations or subtypes, each associated with a specific biological function or state. (C) Pseudotime trajectory indicating gene expression changes over time. (D) Network diagram of cell–cell interactions in the glioma microenvironment. (E): The horizontal axis represents different cell populations or subtypes, such as high NETs microglia, low NETs microglia, endothelial cells, etc. The vertical axis represents signalling pathways (e.g., TNF, VEGF and FGF), with colour intensity indicating signalling strength (darker green signifies higher intensity). (F) Each point represents the contribution intensity of a specific signalling pathway between cell populations. The horizontal and vertical axes represent the signallingsignaling sender and receiver cell populations, respectively. (G) Dot plot of incoming signalling interactions to high NETs microglia.

The dendrogram clusters genes with similar expression patterns (Figure 4A). This UMAP plot visualises the distribution of cells on the basis of their expression profiles, categorised into high and low NETs microglia groups. It shows the clustering of cells, helping to identify differences between these groups (Figure 4B). The pseudotime plot illustrates the progression of cell states along a developmental trajectory. The colour gradient indicates the pseudotime, providing insights into the dynamic changes in gene expression as cells transition through different states (Figure 4C). This network diagram represents the interactions between different cell types, such as microglia, endothelial cells, macrophages and others. The thickness of the lines indicates the strength of interactions, highlighting key communication pathways within the tumour microenvironment (Figure 4D). This heatmap shows outgoing and incoming signalling patterns for various cell types. Each row represents a signalling pathway (e.g., MIF, PTN, GAL1), whereas columns represent different cell types. The colour intensity indicates the relative strength of signalling, providing insights into intercellular communication (Figure 4E). This dot plot visualises the outgoing signalling interactions from high and low NETs microglia to other cell types. The size of the dots represents the significance of the interaction, whereas the colour indicates the strength of the signalling pathway (Figure 4F). Similar to panel F, this dot plot illustrates incoming signalling interactions to high and low NETs microglia from other cell types. The dot size reflects the significance, and the colour represents the strength of the signalling (Figure 4G).

### 3.4 Machine Learning Models and Their Application in Predicting Patient Outcomes in GBM

**FIGURE 5:**
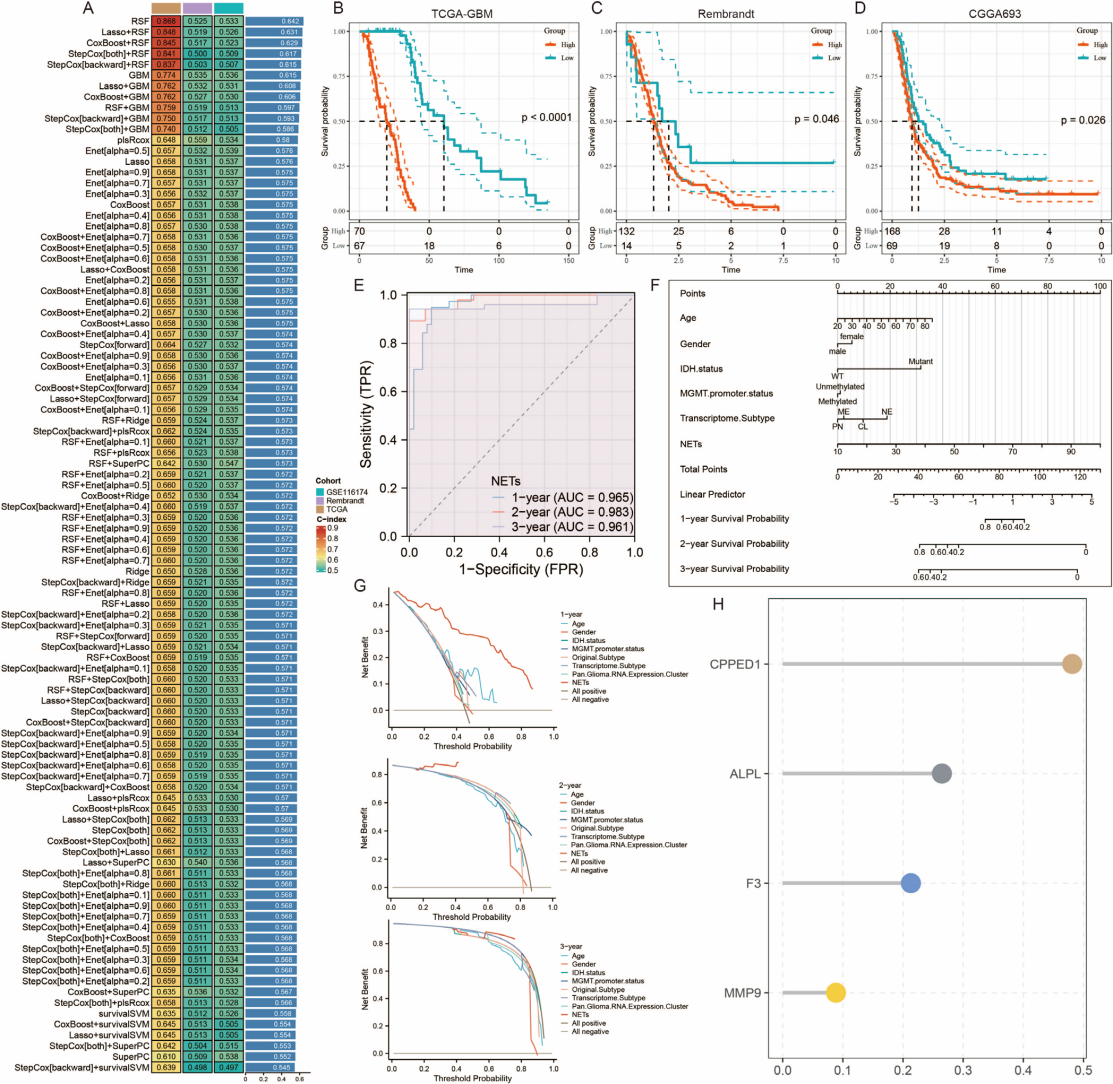
Machine learning models and their application in predicting patient outcomes in GBM. (A) Heatmap comparing the performance of various predictive models for glioma survival. (B) Kaplan–Meier survival curve for TCGA‐GBM dataset, stratified by high and low risk groups. (C) Kaplan–Meier survival curve for Rembrandt dataset, stratified by high and low risk groups. (D) Kaplan–Meier survival curve for CGGA693 dataset, stratified by high and low risk groups. (E) ROC curve showing the sensitivity and specificity of the NETs model at 1, 2 and 3 years. (F) Nomogram for predicting 1‐, 2‐ and 3‐year survival probabilities based on clinical and molecular features. (G) Decision curve analysis for the NETs model at 1, 2 and 3 years. (H) Dot plot showing the importance of genes CPED1, ALPL, F3 and MMP9 in the predictive model.

This panel displays a heatmap comparing the performance of various machine learning models and feature selection methods in predicting patient outcomes. Each row represents a different model or combination of models, and the columns represent different performance metrics (e.g., AUC, accuracy). The colour gradient indicates the performance level, with higher values representing better performance (Figure 5A). These Kaplan–Meier survival curves show the OS of patients stratified by high and low expression of a particular gene across three different datasets: TCGA‐GBM (5B), Rembrandt (5C) and CGGA693 (5D). The *p*‐values indicate the significance of the differences in survival between the groups, with the number of patients at risk shown below each plot (Figure 5B–D). PredictorThis ROC (receiver operating characteristic) curve evaluates the performance of the NETs predictor in distinguishing between high and low risk of adverse outcomes. The area under the curve (AUC) values for 1‐, 2‐ and 3‐year survival predictions are provided, indicating high predictive accuracy (Figure 5E). This nomogram integrates various clinical and molecular factors (e.g., age, gender, IDH status, MGMT promoter status, transcriptome subtype, NETs) to predict 1‐, 2‐ and 3‐year survival probabilities. Each factor receives a score, and total points predict survival probabilities (Figure 5F). These decision curve analysis plots assess the clinical utility of the NETs predictor for 1‐, 2‐ and 3‐year survival by comparing the net benefits of using the predictor versus other models or no model at different threshold probabilities. The curves demonstrate the added value of the NETs predictor in clinical decision‐making (Figure 5G). This dot plot visualises the importance of specific genes (e.g., CPPED1, ALPL, F3 and MMP9) in the NETs predictor model. The size of the dots represents the relative importance of each gene, whereas the colour indicates the level of significance, highlighting key genes that contribute to the model's predictive power (Figure 5H).

### 3.5 Immune Checkpoint Molecule Expression, Gene Ontology and Pathway Enrichment

**FIGURE 6:**
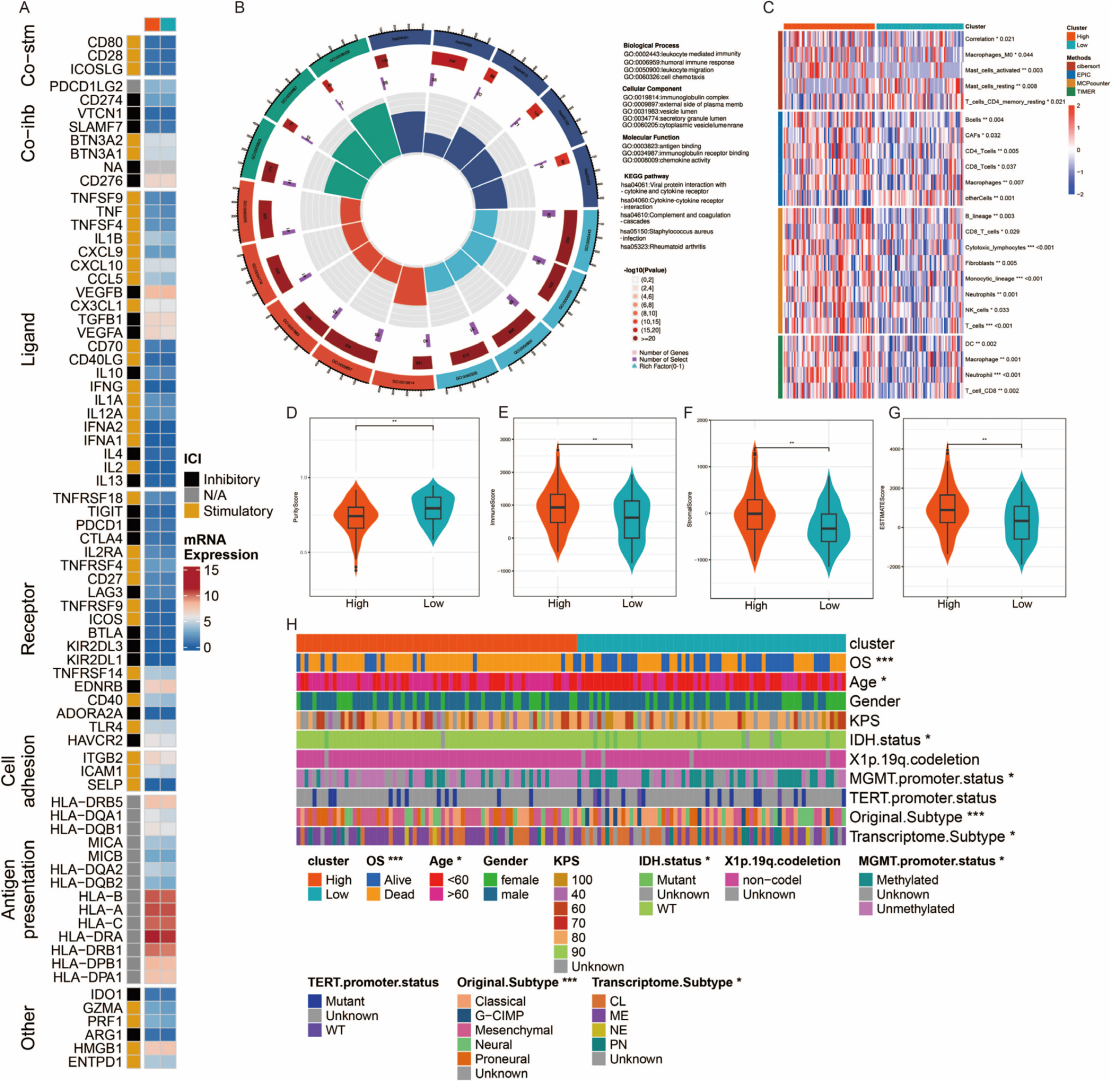
Immune checkpoint molecule expression, gene ontology and pathway enrichment. (A) Heatmap of mRNA expression for immune‐related genes across different categories. (B) Circular plot showing the distribution and interactions of immune checkpoints. (C) Heatmap illustrating the expression of key genes in different sample groups. (D–G) Violin plots comparing expression levels of specific genes between high and low expression groups. (H) Annotation heatmap showing clinical and molecular features, including survival status and genetic subtypes.

This heatmap displays the expression levels of various immune checkpoint molecules across different conditions. Each row represents an immune checkpoint molecule. The ICI (Immune Checkpoint Inhibitor) status is also indicated as inhibitory, stimulatory, or not applicable (N/A) (Figure 6A). This circular plot visualises the enrichment of various GO terms and KEGG pathways for genes associated with different biological processes. The outer ring represents different categories, such as biological processes, cellular components, molecular functions and KEGG pathways. The inner segments show the number of genes associated with each category, with the colour indicating the significance level (adjusted *p*‐value) (Figure 6B). This heatmap displays the expression levels of selected genes across different clusters. Clusters are annotated on the top, and key genes with significant expression differences are labelled (Figure 6C). These violin plots compare the expression levels of specific genes between high and low groups. The width of the plot represents the density of the data points, and the box plot inside shows the median and interquartile range. Significant differences between the groups are indicated by asterisks (*) (Figure 6D–G). This heatmap integrates various clinical and molecular characteristics for each sample. Each row represents a different characteristic, such as cluster, OS, age, gender, Karnofsky Performance Status (KPS), IDH status, 1p/19q codeletion status, MGMT promoter status, TERT promoter status, original subtype and transcriptome subtype. The colour coding indicates different categories or values for each characteristic, providing a comprehensive overview of the patient cohort (Figure 6H).

### 3.6 The Importance of Integrating CNV and Mutation Data in GBM

**FIGURE 7:**
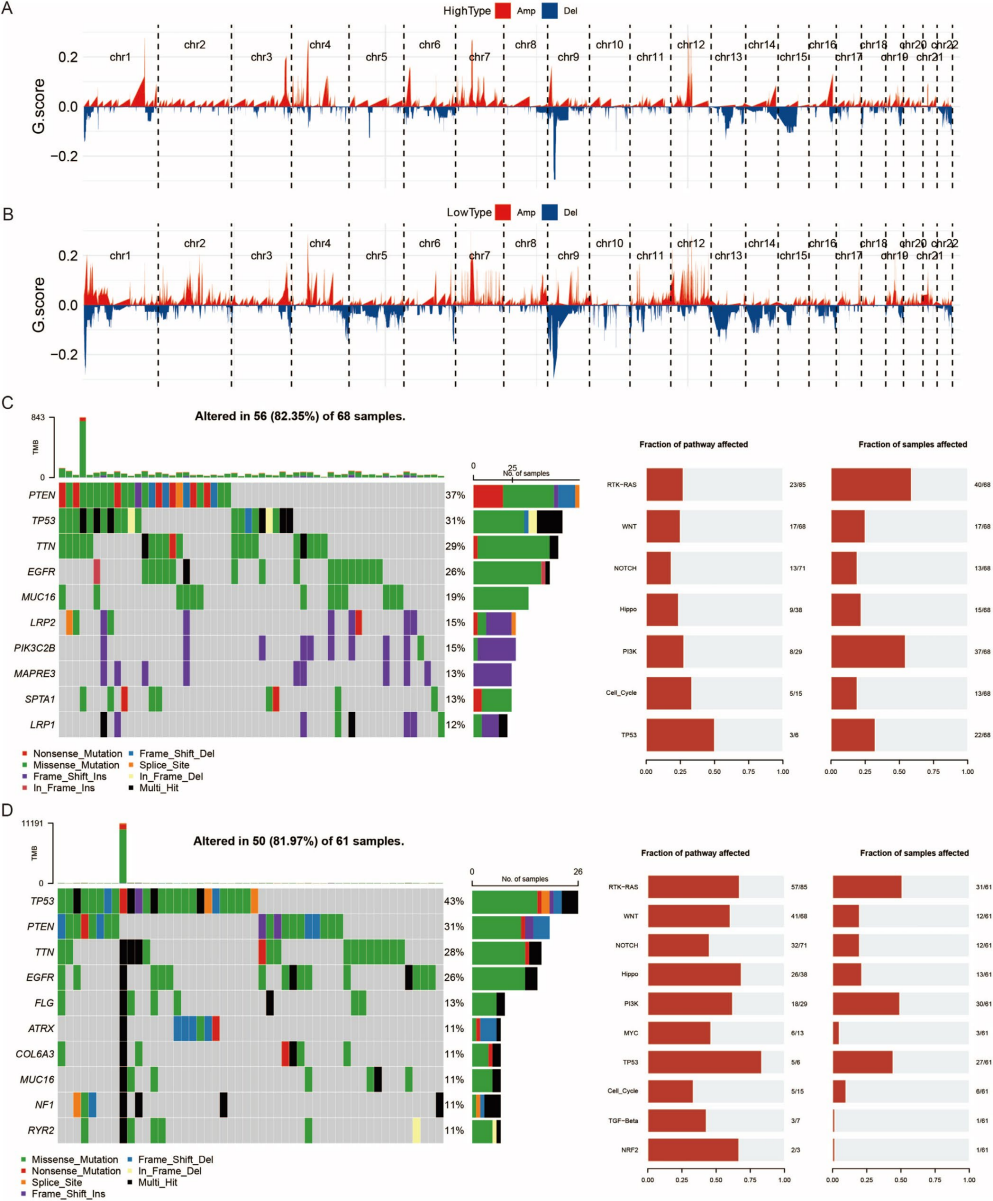
The importance of integrating CNV and mutation data in GBM. (A) Genomic alteration profile for high‐type samples, showing amplifications (red) and deletions (blue) across chromosomes. (B) Genomic alteration profile for low‐type samples, showing amplifications (red) and deletions (blue) across chromosomes. (C) Oncoprint displaying the mutation landscape of key genes in 68 samples, with bar charts indicating the fraction of pathways and samples affected. (D) Oncoprint displaying the mutation landscape of key genes in 61 samples, with bar charts indicating the fraction of pathways and samples affected.

These panels display the genome‐wide copy number variation profiles for two different groups: HighType (Figure 7A) and LowType (Figure 7B). The x‐axis represents chromosomal locations from chromosome 1 to 22, and the y‐axis shows the G score, which indicates the degree of amplification (red) or deletion (blue) for each genomic region. Peaks above the baseline represent amplifications, whereas troughs below the baseline indicate deletions. Key chromosomal regions with significant CNVs are highlighted. The mutation landscapes illustrate the prevalence of mutations in key genes and their impact on critical signalling pathways. The oncoprint visualisations provide a detailed view of the types and frequencies of mutations, whereas the pathway analysis highlights the broader biological implications of these mutations (Figure 7C,D).

### 3.7 Understanding of Glioma Biology and Inform Potential Treatment Strategies

**FIGURE 8:**
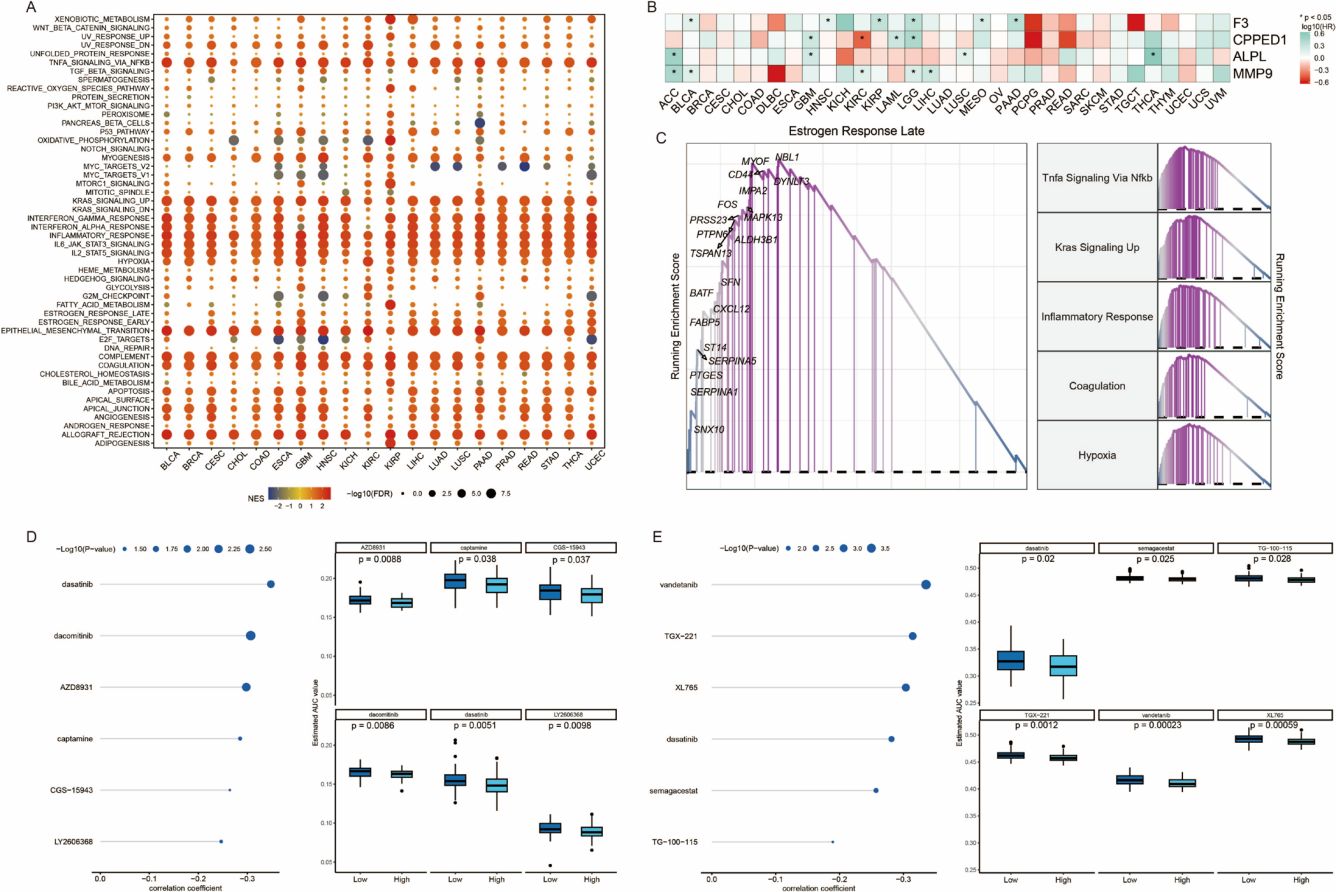
Understanding of glioma biology and inform potential treatment strategies. (A) Dot plot showing enrichment scores for various pathways across different conditions, with dot size indicating significance. (B) Heatmap of gene expression for F3, CPED1, ALPL and MMP9 across multiple cancer types. (C) GSEA plot illustrating enrichment of the oestrogen response late pathway and other key pathways. (D) Dot plot displaying drug sensitivity correlations with specific gene expressions, highlighting significant associations. (E) Box plots comparing expression levels of selected genes between high and low expression groups, with statistical significance indicated.

This dot plot illustrates the enrichment of various biological pathways across different conditions. Each row corresponds to a pathway, and each column represents a condition or dataset. The colour gradient reflects the normalised enrichment score (NES), with red indicating positive enrichment and blue indicating negative enrichment. Dot size denotes the significance level (Figure 8A). The heatmap displays expression levels of specific genes (F3, CPPED1, ALPL and MMP9) across samples or conditions. Each row represents a gene, and each column a sample. The colour gradient shows expression levels, with red for high expression and blue for low. Asterisks mark significant differences (Figure 8B). The GSEA plot highlights the enrichment of the “Estrogen Response Late” gene set. The x‐axis shows the rank order of genes, and the y‐axis indicates the running enrichment score. The curve's peak marks maximum enrichment. Additional plots on the right display enrichment scores for pathways like TNFA signalling, KRAS signalling, inflammatory response, coagulation and hypoxia (Figure 8C). This panel includes a dot plot of drug sensitivity, showing the correlation between gene expression and drug response. Dot size signifies significance level, highlighting drugs with notable associations (Figure 8D). Box plots compare expression levels of specific genes between high and low expression groups. The x‐axis shows the groups, and the y‐axis indicates expression levels. Boxes represent the interquartile range, with the median as a line inside. P‐values denote significant differences between groups (Figure 8E).

### 3.8 A Comprehensive View of the Molecular Interactions and Cellular Contexts

**FIGURE 9:**
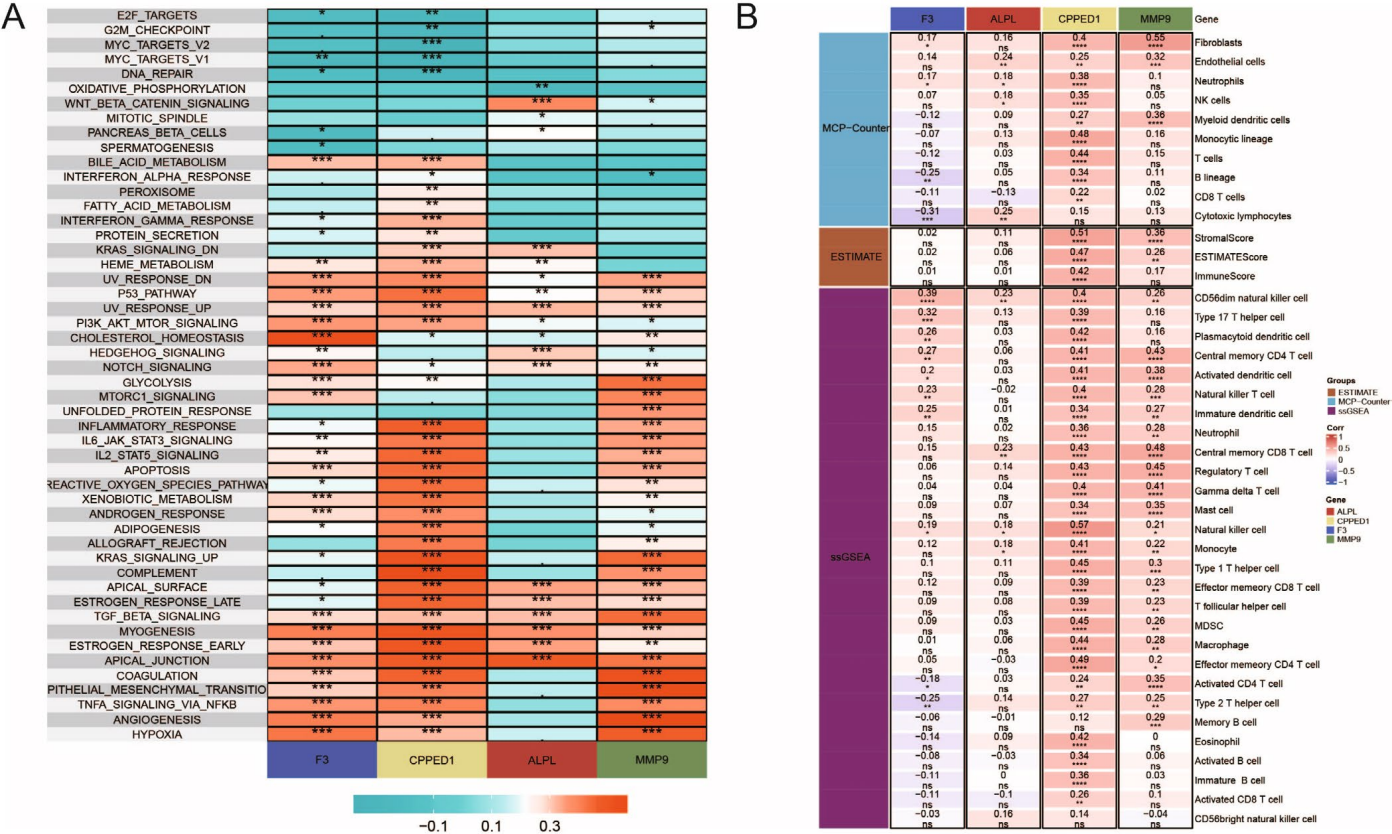
A comprehensive view of the molecular interactions and cellular contexts. (A) Heatmap showing the correlation between gene expression (F3, CPED1, ALPL and MMP9) and various biological pathways. (B) Heatmap displaying the association between gene expression (F3, ALPL, CPED1 and MMP9) and immune cell infiltration scores.

This heatmap shows the correlation between gene expression and the enrichment of various biological pathways. Each row represents a pathway, and each column represents a gene (F3, CPPED1, ALPL and MMP9). The colour gradient indicates the correlation coefficient, with red representing positive correlation and teal representing negative correlation. Asterisks indicate the significance level, with more asterisks denoting higher significance (Figure 9A). This heatmap displays the correlation coefficients between the expression of specific genes (F3, CPPED1, ALPL and MMP9) and various cell types or scores from different computational methods (MCP‐Counter, ESTIMATE, ssGSEA). Each row represents a cell type or score, and each column represents a gene. The colour gradient indicates the correlation strength, with red representing positive correlation and blue representing negative correlation. The numbers inside the cells show the actual correlation values (Figure 9B).

### 3.9 Detailed Analysis of F3 Gene Expression and Its Spatial Distribution Within a Tissue Section

**FIGURE 10:**
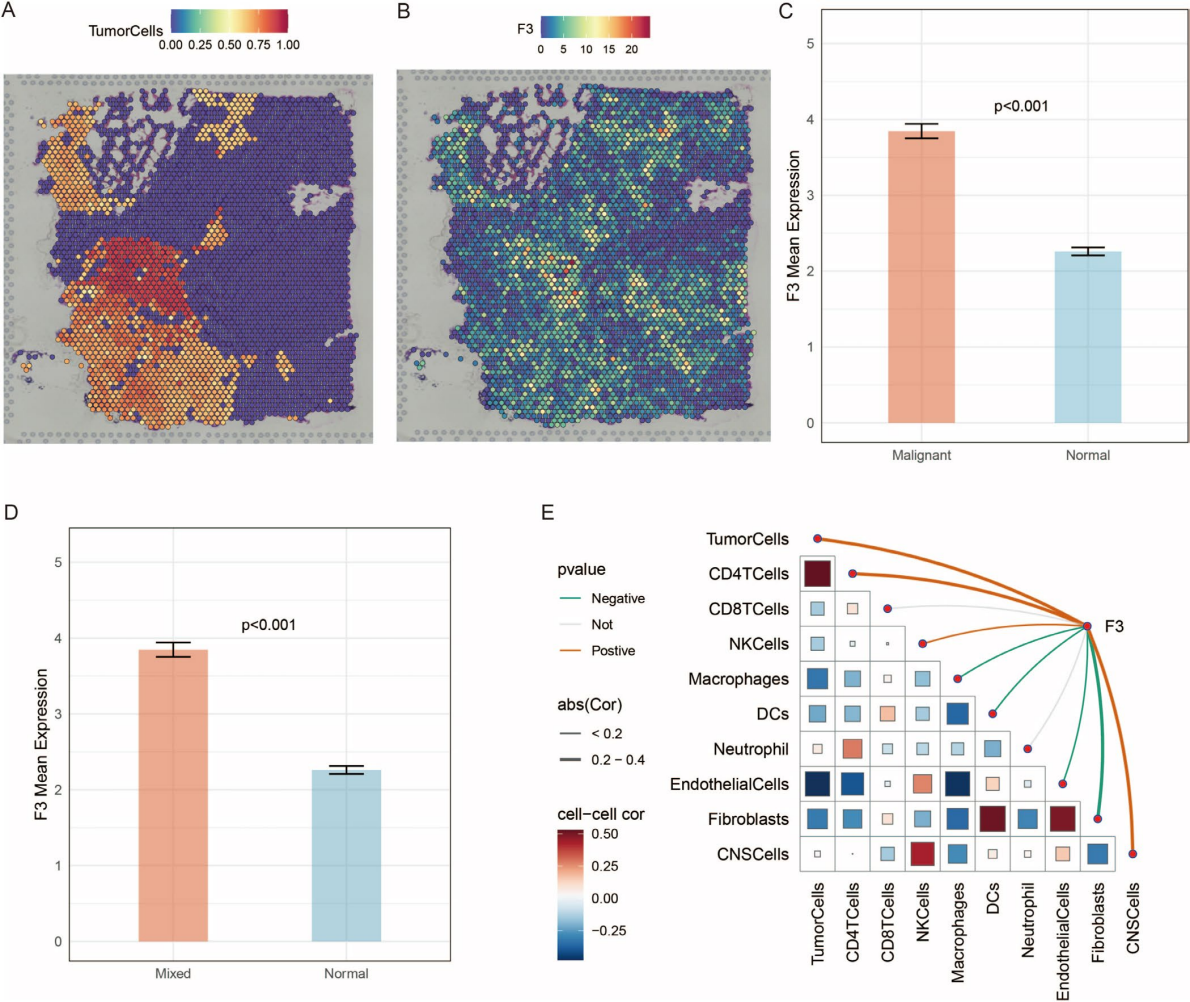
Detailed analysis of F3 gene expression and its spatial distribution within a tissue section. (A) Spatial distribution of tumour cells, with colour gradient representing the proportion of tumour cells (0.00–1.00). (B) F3 gene expression levels, with colour gradient indicating expression intensity (0–20). (C) Bar graph comparing mean F3 expression between malignant and normal tissues. (D) Bar graph comparing mean F3 expression between mixed and normal tissues. (E) Correlation matrix showing associations between F3 expression and various cell types.

The heatmap represents the density of tumour cells across the tissue section. The colour gradient ranges from purple (low density) to red (high density), indicating areas with varying concentrations of tumour cells (Figure 10A). This heatmap displays the expression levels of the F3 gene across the same tissue section. The colour gradient ranges from blue (low expression) to red (high expression), highlighting regions with different levels of F3 expression (Figure 10B). The box plot shows a significant difference (*p* < 0.001) in F3 expression between malignant and normal tissues, with higher expression in malignant tissues (Figure 10C). Similarly, this box plot illustrates a significant difference (*p* < 0.001) in F3 expression between mixed (tumour and normal) and normal tissues, with higher expression in the mixed group (Figure 10D). This matrix visualises the correlation between F3 expression and various cell types within the tissue (Figure 10E).

### 3.10 Association Between F3 Gene Expression and Coronary Heart Disease

**FIGURE 11:**
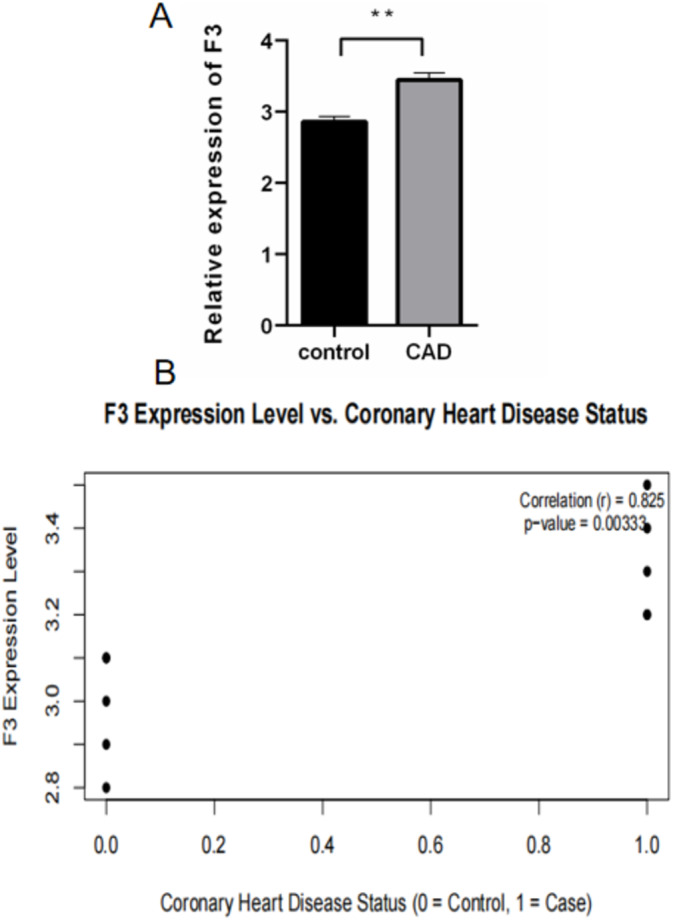
Association Between F3 Gene Expression and Coronary Heart Disease. (A) Bar graph showing the relative expression of the F3 gene in control versus coronary artery disease (CAD) groups. The expression is significantly higher in the CAD group compared to the control group (*p* < 0.01). (B) Scatter plot illustrating the correlation between F3 expression levels and coronary heart disease status. A strong positive correlation is observed (*r* = 0.825), with a statistically significant *p*‐value of 0.0033.

Research results indicate that F3 gene expression levels are significantly higher in CHD patients compared to the control group. Statistical analysis shows a significant positive correlation between F3 gene expression and CHD status, with a correlation coefficient of 0.825 and a *p*‐value of 0.0033, indicating statistical significance. This suggests that the F3 gene may play an important role in the onset or progression of CHD (Figure 11A,B).

## 4 Discussion

This study revealed a potential association between CHD and glioma through machine learning and single‐cell expression analysis, thereby confirming the complex genetic link between the two. MR analysis identified a number of genetic variants that were significantly associated with both CHD and glioma. In particular, the expression of the F3 gene showed a key correlation between the two diseases. Machine learning has become an indispensable tool in the analysis of large‐scale genomic data, allowing for the identification of intricate patterns that may not be discernible through conventional methods [14].

Although CHD and glioma are ostensibly distinct diseases affecting the cardiovascular and neurological systems, respectively, the results of this study indicate that they may share some common molecular mechanisms. In particular, following a joint analysis of data from CHD and gliomas, the results of MR suggest that genetic variants in gliomas may increase the risk of developing CHD to some extent. This association provides a theoretical basis for further studies of disease co‐morbidity, suggesting that CHD and gliomas may interact in disease development through a common genetic network or signalling pathway.

In this study, machine learning algorithms were instrumental in processing and analysing genome‐wide association study (GWAS) data, leading to the identification of genetic variants associated with glioma. These algorithms can handle the high dimensionality and complexity of genomic data, efficiently uncovering potential biomarkers and therapeutic targets.

The application of machine learning in single‐cell RNA sequencing (scRNA‐seq) further enhanced our understanding of gene expression dynamics within glioma. By analysing the expression patterns of the identified genetic variants across different cell types, we were able to pinpoint specific cellular contexts in which these genes play critical roles. This is particularly important in glioma, where the tumour microenvironment is highly heterogeneous and influences disease progression and treatment response [15].

By analysing the expression patterns of the identified genetic variants across different cell types, we were able to pinpoint specific cellular contexts in which these genes play critical roles. This is particularly important in glioma, where the tumour microenvironment is highly heterogeneous and influences disease progression and treatment response.

Our analysis revealed that the F3 gene is highly expressed in glioma, suggesting its potential role in tumorigenesis. The F3 gene, also known as CD142, is commonly associated with thrombosis and vascular endothelial function [16] and is a potential risk factor for CHD [17, 18]. It has been shown that CD142^+^ MSCs are highly secreted and can protect cardiomyocytes during myocardial infarction [19]. There is also evidence that these CD142^+^ cells are enriched in atherosclerotic platelets, possibly associated with an inflammatory response [20]. On the other hand, high F3 gene expression in the glioma microenvironment may influence tumour angiogenesis and cellular interactions in the tumour microenvironment, thereby promoting glioma progression [21, 22]. In addition, the importance of the F3 gene in coronary artery disease may also apply to glioma, and by affecting the inflammatory response and vascular endothelial function, it may play an important role in tumour angiogenesis and tumour cell growth [23]. Thus, the role of the F3 gene in CHD and glioma may provide new perspectives for understanding the association between these two diseases.

In this study, our results indicated the correlation between F3 expression and various cell types within the tissue highlights its involvement in the tumour microenvironment, possibly influencing cell–cell interactions and signalling pathways critical for glioma development [24]. Several previous studies have also suggested that the F3 gene may be strongly correlated with neutrophil extracellular traps and, through the immune response, with the prognosis of glioma [25]. In addition to glioma, F3 is widely involved in the development of cancers such as multiple myeloma [26], pancreatic cancer [27] and colorectal cancer [28]. The integration of genetic inference, machine learning and single‐cell expression analysis provides a comprehensive framework for identifying and validating novel therapeutic targets in glioma. By establishing causal relationships using MR methods, we can prioritise genetic targets that have a direct impact on disease phenotypes, thereby guiding the development of more effective and personalised treatment strategies.

Although this study provides valuable insights, several limitations should be acknowledged. The robustness of our findings is limited by the sample size and diversity of the GWAS and scRNA‐seq datasets. Larger, more diverse cohorts are needed to validate our results and ensure their generalizability across different populations. Single‐cell RNA sequencing data can be affected by technical variability, including batch effects and differences in cell capture efficiency. Although we employed normalisation techniques, these factors could still influence the results. Although we identified potential genetic targets and their expression patterns, functional validation through in vivo studies and clinical trials is necessary to confirm their roles in glioma and CHD. Our in vitro experiments on F3 provide preliminary evidence, but further investigation is required. Integrating multi‐omic data to provide a holistic view of the molecular mechanisms driving glioma and CHD, facilitating the identification of novel therapeutic targets and biomarkers.

### 4.1 Limitations

Our research findings may be limited by the sample size and diversity of GWAS and scRNA seq datasets. To validate these findings, research needs to be conducted in larger and more diverse cohorts to ensure universal applicability of the results across different populations. Single cell RNA sequencing data may be affected by technological variability, including batch effects and differences in cell capture efficiency. Although we have adopted standardised techniques, these factors may still affect the results. Although we have identified potential genetic targets and expression patterns through machine learning algorithms and single‐cell expression analysis, functional validation through in vivo studies and clinical trials is necessary to confirm their roles in glioma and CHD. Future research should focus on validating the role of F3 gene in glioma and CHD through in vivo models and clinical trials. In addition, investigate the expression patterns of F3 gene in different cell types.

## 5 Conclusion

In conclusion, this study provides novel insights into the potential genetic association between CHD and glioma, with a particular focus on the role of the F3 gene. Through MR and single‐cell expression analysis, we identified F3 as a critical gene highly expressed in both CHD and glioma contexts, suggesting its involvement in angiogenesis, endothelial function and inflammatory response. The integration of machine learning with genomic and transcriptomic data has enabled the identification of F3 as a promising therapeutic target, offering a foundation for future studies to explore targeted interventions for these complex diseases. This study underscores the importance of investigating shared molecular mechanisms across diseases and provides a pathway for developing precision medicine strategies that address the unique characteristics of both CHD and glioma. Further research, including in vivo studies and clinical trials, is essential to validate these findings and explore the clinical applicability of targeting F3 in the treatment of CHD and glioma. We also urge researchers to focus on the risk of CHD in glioma patients with high F3 gene expression.

## Author Contributions

**Youfu He:** conceptualization (equal), data curation (equal), writing – original draft (equal). **Ganhua You:** formal analysis (equal), validation (equal). **Yu Zhou:** data curation (equal), formal analysis (equal), resources (equal). **Liqiong Ai:** methodology (equal), supervision (equal). **Wei Liu:** formal analysis (equal), validation (equal). **Xuantong Meng:** methodology (equal), visualization (equal). **Qiang Wu:** funding acquisition (equal), project administration (equal), writing – review and editing (equal).

## Ethics Statement

The ethical review of This study was approved by the Ethics Committee of the Guizhou Provincial People's Hospital (Lun Audit (Research) 2024‐523).

## Conflicts of Interest

The authors declare no conflicts of interest.

## Supporting information


**Table S1.** Results of SMR analyses.

## Data Availability

The datasets used during the current study are available from the corresponding author on reasonable request.

## References

[1] A. Sarantopoulos , C. Ene , and E. Aquilanti , “Therapeutic Approaches to Modulate the Immune Microenvironment in Gliomas,” npj Precision Oncology 8, no. 1 (2024): 241. 39443641 10.1038/s41698-024-00717-4 PMC11500177

[2] W. T. Sun , J. Y. Du , J. Wang , Y. L. Wang , and E. D. Dong , “Potential Preservative Mechanisms of Cardiac Rehabilitation Pathways on Endothelial Function in Coronary Heart Disease,” Science China. Life Sciences 68 (2024): 158–175. 39395086 10.1007/s11427-024-2656-6

[3] S. Haase , S. Carney , M. L. Varela , et al., “Epigenetic Reprogramming in Pediatric Gliomas: From Molecular Mechanisms to Therapeutic Implications,” Trends Cancer 10 (2024): 1147–1160. 39394009 10.1016/j.trecan.2024.09.007 PMC11631670

[4] J. S. Bhardwaj , S. Paliwal , G. Singhvi , and R. Taliyan , “Immunological Challenges and Opportunities in Glioblastoma Multiforme: A Comprehensive View From Immune System Lens,” Life Sciences 357 (2024): 123089. 39362586 10.1016/j.lfs.2024.123089

[5] C. Madaudo , G. Bono , A. Ortello , et al., “Dysfunctional High‐Density Lipoprotein Cholesterol and Coronary Artery Disease: A Narrative Review,” Journal of Personalized Medicine 14, no. 9 (2024): 996. 39338250 10.3390/jpm14090996 PMC11432852

[6] D. McGonagle and S. Giryes , “An Immunology Model for Accelerated Coronary Atherosclerosis and Unexplained Sudden Death in the COVID‐19 Era,” Autoimmunity Reviews 23, no. 11 (2024): 103642. 39313122 10.1016/j.autrev.2024.103642

[7] F. D. Testai , P. B. Gorelick , P. Y. Chuang , et al., “Cardiac Contributions to Brain Health: A Scientific Statement From the American Heart Association,” Stroke 55, no. 12 (2024): e425–e438. 39387123 10.1161/STR.0000000000000476

[8] L. Liu , B. Davidorf , P. Dong , A. Peng , Q. Song , and Z. He , “Decoding the Mosaic of Inflammatory Bowel Disease: Illuminating Insights With Single‐Cell RNA Technology,” Computational and Structural Biotechnology Journal 23 (2024): 2911–2923. 39421242 10.1016/j.csbj.2024.07.011 PMC11485491

[9] A. Sarantopoulos , C. Mastori Kourmpani , A. L. Yokarasa , et al., “Artificial Intelligence in Infectious Disease Clinical Practice: An Overview of Gaps, Opportunities, and Limitations,” Tropical Medicine and Infectious Disease 9, no. 10 (2024): 228. 39453255 10.3390/tropicalmed9100228 PMC11511260

[10] J. Sharma , V. Jangale , A. K. Swain , and P. Yadav , “An Optimized Instrument Variable Selection Approach to Improve Causality Estimation in Association Studies,” Scientific Reports 14, no. 1 (2024): 22781. 39354059 10.1038/s41598-024-73970-z PMC11445377

[11] V. W. Skrivankova , R. C. Richmond , B. A. R. Woolf , et al., “Strengthening the Reporting of Observational Studies in Epidemiology Using Mendelian Randomisation (STROBE‐MR): Explanation and Elaboration,” BMJ 375 (2021): n2233. 34702754 10.1136/bmj.n2233 PMC8546498

[12] K. Li , J. Fan , X. Qin , and Q. Wei , “Novel Therapeutic Compounds for Prostate Adenocarcinoma Treatment: An Analysis Using Bioinformatic Approaches and the CMap Database,” Medicine 99, no. 51 (2020): e23768. 33371142 10.1097/MD.0000000000023768 PMC7748316

[13] Z. Lu , M. Chen , Y. Zong , C. Huang , X. Li , and P. Zhou , “Sensitivity Analysis of CMAP Scan Step Index to Different Stimulation Parameters and Examination of Muscles Affected by Spinal Cord Injury,” IEEE Transactions on Biomedical Engineering 70, no. 10 (2023): 2834–2840. 37756167 10.1109/TBME.2023.3266327 PMC11057332

[14] H. S. Luu , “Laboratory Data as a Potential Source of Bias in Healthcare Artificial Intelligence and Machine Learning Models,” Annals of Laboratory Medicine 45 (2024): 12–21. 39444135 10.3343/alm.2024.0323 PMC11609702

[15] L. Noor , A. Upadhyay , and V. Joshi , “Role of T Lymphocytes in Glioma Immune Microenvironment: Two Sides of a Coin,” Biology 13, no. 10 (2024): 846. 39452154 10.3390/biology13100846 PMC11505600

[16] E. Teer , D. E. Joseph , N. Driescher , et al., “HIV and Cardiovascular Diseases Risk: Exploring the Interplay Between T‐Cell Activation, Coagulation, Monocyte Subsets, and Lipid Subclass Alterations,” American Journal of Physiology. Heart and Circulatory Physiology 316, no. 5 (2019): H1146–H1157. 30768357 10.1152/ajpheart.00797.2018

[17] G. Chiva‐Blanch , K. Laake , P. Myhre , et al., “Platelet‐, Monocyte‐Derived and Tissue Factor‐Carrying Circulating Microparticles Are Related to Acute Myocardial Infarction Severity,” PLoS One 12, no. 2 (2017): e0172558. 28207887 10.1371/journal.pone.0172558 PMC5313202

[18] G. Chiva‐Blanch , V. Bratseth , V. Ritschel , et al., “Monocyte‐Derived Circulating Microparticles (CD14(+), CD14(+)/CD11b(+) and CD14(+)/CD142(+)) Are Related to Long‐Term Prognosis for Cardiovascular Mortality in STEMI Patients,” International Journal of Cardiology 227 (2017): 876–881. 27915085 10.1016/j.ijcard.2016.11.302

[19] X. Wang , C. Yang , X. Ma , et al., “A Division‐Of‐Labor Mode Contributes to the Cardioprotective Potential of Mesenchymal Stem/Stromal Cells in Heart Failure Post Myocardial Infarction,” Frontiers in Immunology 15 (2024): 1363517. 38562923 10.3389/fimmu.2024.1363517 PMC10982400

[20] A. C. Bashore , H. Yan , C. Xue , et al., “High‐Dimensional Single‐Cell Multimodal Landscape of Human Carotid Atherosclerosis,” Arteriosclerosis, Thrombosis, and Vascular Biology 44, no. 4 (2024): 930–945. 38385291 10.1161/ATVBAHA.123.320524 PMC10978277

[21] W. Xu , B. Chen , D. Ke , and X. Chen , “CD142 Plays a Key Role in the Carcinogenesis of Gastric Adenocarcinoma by Inhibiting BCL2‐Dependent Autophagy,” Biochemistry and Cell Biology 100, no. 1 (2022): 17–27. 34289309 10.1139/bcb-2021-0144

[22] Q. Gao , Z. Chen , Y. He , et al., “CD142 Plays an Important Role in the Mobility of Colorectal Cancer Cells,” Bioscience, Biotechnology, and Biochemistry 84, no. 9 (2020): 1856–1860. 32471327 10.1080/09168451.2020.1772039

[23] H. M. Jeon , J. Y. Kim , H. J. Cho , et al., “Tissue Factor Is a Critical Regulator of Radiation Therapy‐Induced Glioblastoma Remodeling,” Cancer Cell 41, no. 8 (2023): 1480–1497. 37451272 10.1016/j.ccell.2023.06.007 PMC10530238

[24] F. Racine , S. Soudet , M. A. Sevestre , A. Galmiche , and Z. Saidak , “The Coagulome of Oral Squamous Cell Carcinoma: Examining the Role and Regulation of Coagulation in Oral Cancers Using a Systems Approach,” Current Opinion in Otolaryngology & Head and Neck Surgery 31, no. 2 (2023): 73–77. 36912218 10.1097/MOO.0000000000000870

[25] G. Sun and W. Liu , “The Neutrophil Extracellular Traps‐Related Gene Signature Predicts the Prognosis of Glioblastoma Multiforme,” Folia Neuropathologica 62, no. 1 (2024): 59–75. 38174685 10.5114/fn.2023.132980

[26] G. Cesarman‐Maus , E. Braggio , H. Maldonado , and R. Fonseca , “Absence of Tissue Factor Expression by Neoplastic Plasma Cells in Multiple Myeloma,” Leukemia 26, no. 7 (2012): 1671–1674. 22333877 10.1038/leu.2012.43

[27] M. Li , W. Ding , Y. Wang , Y. Ma , and F. Du , “Development and Validation of a Gene Signature for Pancreatic Cancer: Based on Inflammatory Response‐Related Genes,” Environmental Science and Pollution Research International 30, no. 7 (2023): 17166–17178. 36192587 10.1007/s11356-022-23252-w

[28] A. A. Soos , A. Kelemen , A. Orosz , et al., “High CD142 Level Marks Tumor‐Promoting Fibroblasts With Targeting Potential in Colorectal Cancer,” International Journal of Molecular Sciences 24, no. 14 (2023): 11585. 37511344 10.3390/ijms241411585 PMC10381019

